# Quantitative MRI-based radiomics analysis identifies blood flow feature associated to overall survival for rectal cancer patients

**DOI:** 10.1038/s41598-023-50966-9

**Published:** 2024-01-02

**Authors:** Franziska Knuth, Fariba Tohidinezhad, René M. Winter, Kine Mari Bakke, Anne Negård, Stein H. Holmedal, Anne Hansen Ree, Sebastian Meltzer, Alberto Traverso, Kathrine Røe Redalen

**Affiliations:** 1 Department of Physics, Norwegian University of Science and Technology, Høgskoleringen 5, 7491 Trondheim, Norway; 2 Department of Radiation Oncology (Maastro Clinic), School for Oncology and Developmental Biology (GROW), Maastricht University Medical Center, Maastricht, The Netherlands; 3 Department of Oncology, Akershus University Hospital, Lørenskog, Norway; 4 Institute of Clinical Medicine, University of Oslo, Oslo, Norway; 5 Department of Radiology, Akershus University Hospital, Lørenskog, Norway

**Keywords:** Prognostic markers, Cancer imaging, Rectal cancer, Biological physics

## Abstract

Radiomics objectively quantifies image information through numerical metrics known as features. In this study, we investigated the stability of magnetic resonance imaging (MRI)-based radiomics features in rectal cancer using both anatomical MRI and quantitative MRI (qMRI), when different methods to define the tumor volume were used. Second, we evaluated the prognostic value of stable features associated to 5-year progression-free survival (PFS) and overall survival (OS). On a 1.5 T MRI scanner, 81 patients underwent diagnostic MRI, an extended diffusion-weighted sequence with calculation of the apparent diffusion coefficient (ADC) and a multiecho dynamic contrast sequence generating both dynamic contrast-enhanced and dynamic susceptibility contrast (DSC) MR, allowing quantification of K^trans^, blood flow (BF) and area under the DSC curve (AUC). Radiomic features were extracted from T2w images and from ADC, K^trans^, BF and AUC maps. Tumor volumes were defined with three methods; machine learning, deep learning and manual delineations. The interclass correlation coefficient (ICC) assessed the stability of features. Internal validation was performed on 1000 bootstrap resamples in terms of discrimination, calibration and decisional benefit. For each combination of image and volume definition, 94 features were extracted. Features from qMRI contained higher prognostic potential than features from anatomical MRI. When stable features (> 90% ICC) were compared with clinical parameters, qMRI features demonstrated the best prognostic potential. A feature extracted from the DSC MRI parameter BF was associated with both PFS (*p* = 0.004) and OS (*p* = 0.004). In summary, stable qMRI-based radiomics features was identified, in particular, a feature based on BF from DSC MRI was associated with both PFS and OS.

## Introduction

Worldwide, 1.9 million men and women were diagnosed with colorectal cancer (CRC) in 2020; of which about 30% were in the rectal anatomic site^1^. CRC is common in both sexes and in all adult ages, but the incidence is rising significantly from the age of 50^2^. Although last decades’ major improvements in neoadjuvant chemo- and radiotherapy (CRT) for locally advanced rectal cancer as well as optimized surgical techniques have resulted in improved local recurrence rates^3^, around 40% of these patients develop metastatic disease^4^, which is the primary cause of death. Therefore, precise and early detection of the patients who are at risk of having aggressive disease would help in order to offer a personalized treatment plan for each patient.

There has been a huge expansion in the use of medical imaging in oncology during the last decade. The technological advances in magnetic resonance imaging (MRI) has resulted in new opportunities within diagnostic radiology and imaging biomarker development. MRI with high-resolution T2-weighted sequences is mandatory in the diagnostic work-up of rectal cancer and provides zonal anatomy and a detailed evaluation of local disease extension, regional metastasis and general anatomy^5^. In addition, functional MRI comprises sequences reflecting microenvironmental properties such as tumor oxygenation (by blood level-dependent oxygenation (BOLD) MRI)^6,7^, tissue structure (by diffusion-weighted (DW) MRI)^8^ and tumor vascularity (by dynamic contrast-enhanced (DCE) MRI and dynamic susceptibility contrast (DSC) MRI)^9–11^. Given the advances in recent years, it is expected that functional MR will be of increasing importance for diagnosis and response evaluation in patients with rectal cancer, in order to achieve more individualized and optimized cancer treatment^12,13^.

Embedded in functional images is valuable quantitative information that not only can be used for diagnostics, but also to support treatment decisions and outcome predictions and evaluations^13^. We have previously shown that quantitative MRI (qMRI) parameters contain valuable prognostic information for rectal cancer patients. An oxygenation-related parameter, denoted as tumor R2* peak value, from DSC MRI, identified patients with malignant lymph nodes^9^. More recently, we showed that low tumor blood flow (BF) quantified from DSC MRI was significantly associated with short progression free survival (PFS) and overall survival (OS)^11^. These results were based on identifying median values of the qMRI parameters in heterogeneous tumors and to the best of our knowledge, qMRI measures capturing the tumor heterogeneity have not been previously investigated in rectal cancer.

Radiomics aims to address this topic by objectively quantifying information in the images through numerical metrics known as features^14,15^. Such features may, when analyzed using machine learning algorithms due to their high dimensionality, contain potentially useful predictive and/or prognostic potential capturing tumor heterogeneity. The features can also be developed into signatures, and enable an imaging phenotype of the disease which can be considered as a step towards personalized cancer treatment.

The procedures for extraction and analysis of radiomics features from medical images must be automized and robust with minimal manual input being required^16^. A main task to automize is the delineation of the tumor volume, which is the input to the radiomics analysis. In a previous study, it was identified that both traditional machine learning algorithms^17^ and deep-learning (DL) networks^18^ based on MRI information are able to segment the tumor either semi- or fully automatic, and the results have shown high performance compared to the manual delineations performed by radiologists. However, the variability between automatic and manual delineations remains a challenging issue, and it is not yet fully investigated how this variability will affect the selection and prognostic potential of the extracted radiomics features.

Hence, the first objective of this study was to investigate the stability of the MRI-based radiomics features in our rectal cancer cohort of 81 patients when generating features from both anatomical MRI and qMRI, using three different methods to define the tumor volume; machine learning, deep learning and manual delineations. The second objective was to evaluate the prognostic value of the identified stable radiomics features by assessing the features’ association to the patients’ 5-year PFS and OS.

## Materials and methods

**Figure 1:**
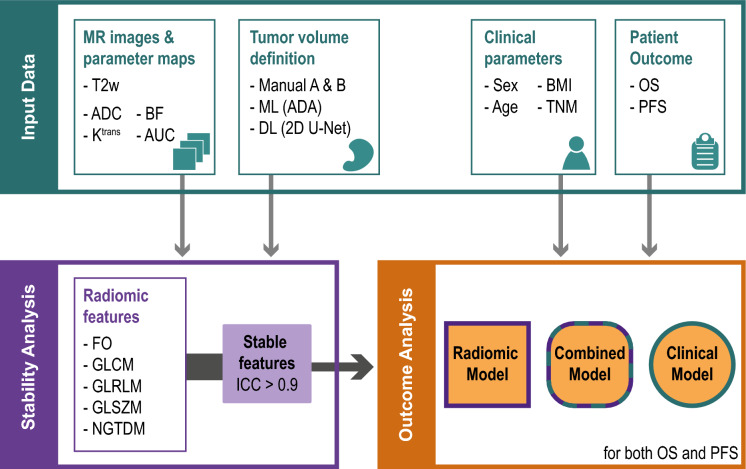
Flowchart illustrating the input to the radiomics analysis consisting of different MRI parameters and different methods to define the tumor volume, before radiomics features were extracted. The stable radiomics features having an interclass correlation coefficient (ICC) above 0.9 were used in outcome analysis (5-year overall survival (OS) and progression-free survival (PFS)), where a model with the radiomics features was compared to a clinical model, but also to a combined model with both clinical and radiomics features. ADC = apparent diffusion coefficient, ADA = adaptive boosting, AUC = area under the curve, BF = blood flow, BMI = body mass index, DL = deep learning, FO = first order, GLCM = gray level cooccurrence matrix, GLDM = gray level dependence matrix, GLRLM = gray level run length matrix, GLSZM = gray level size zone matrix, K^trans^ = plasma transfer constant, ML = machine learning, NGTDM = neighboring gray tone difference matrix, TNM = tumor node metastasis, T2w = T2-weighted MRI.

Figure 1 provides a flowchart detailing the input of MRI parameters and tumor volumes to the radiomics model as well as how radiomics features were extracted and used in outcome analysis together with clinical parameters.

### Patient cohort

**Figure 2:**
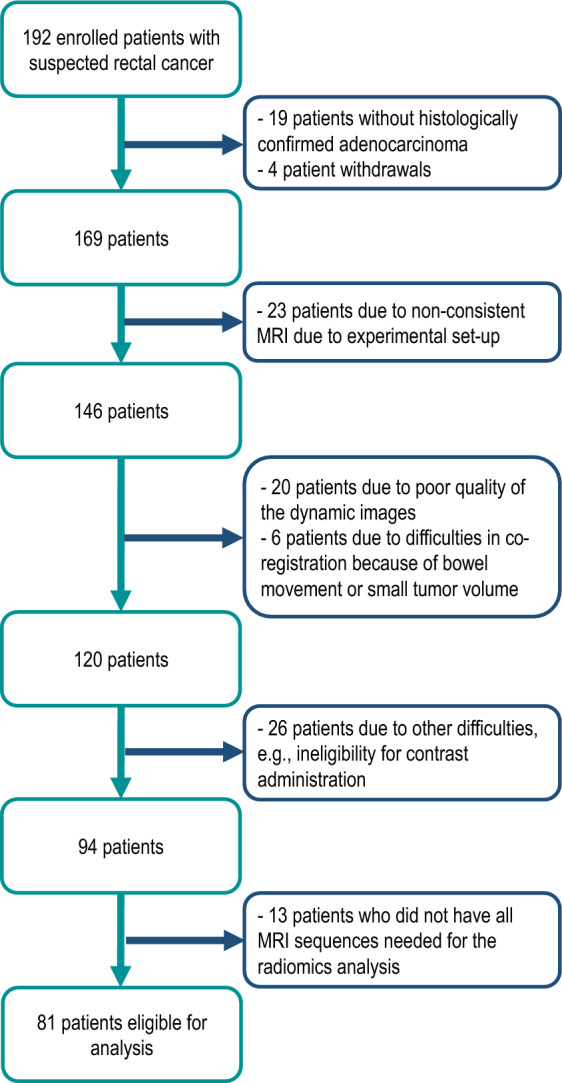
Flowchart of the number of patients eligible for analysis.

Patients included in this analysis were part of the prospective observational OxyTarget study (NCT01816607). The study was performed in accordance with the Helsinki Declaration, and written informed consent was obtained from all participants. Approval was obtained from the Institutional Review Board and the Regional Committee for Medical and Health Research Ethics. Between October 2013 and December 2017, a total of 192 patients with suspected rectal cancer were consecutively enrolled. Eligible participants were older than 18 years and did not have any previous treatment for rectal cancer. Routine and study-specific MRI sequences were carried out at baseline before treatment. For patients receiving neoadjuvant treatment (radiotherapy and/or chemotherapy), a second MRI was performed after treatment completion. Selection of patients for neoadjuvant treatment was determined by the multidisciplinary team, applying the 2013 ESMO Clinical Practice Guidelines^19^ and according to the imaging updates detailed in the 2017 version^20^. Diagnostic T and N stages (mrTN) were determined using at the diagnostic MRI. The histopathological assessment (pTN or ypTN stage) was performed by experienced pathologists after surgery. Distant metastasis (M) was detected with routine thoracoabdominal computed tomography (CT) as recommended by the national follow-up program for colorectal cancer or with individual, supplemental examinations due to clinical suspicion. All patients are followed for five years, the last censoring date was January 31^st^, 2022. No patients were lost to follow-up. The PFS was calculated from study enrolment to first progression (local recurrence, metastasis, or death) or patient censoring due to reaching the maximum follow-up time of 5 years. The OS was calculated from enrolment to death or patient censoring. For the present study, we included data from patients who were enrolled in a previous study on automatic tumor segmentation. Figure 2 shows a flowchart of the number of patients eligible for analysis. Table 1 summarizes the patient characteristics. For the stability analysis of radiomics features, all 81 patient datasets were used. For the subsequent statistical analysis to identify whether clinical and/or stable radiomics features can predict PFS or OS, 19 patients who presented distant metastases at the time of primary diagnosis were excluded.

**Table 1:** Patient characteristics.

Parameter	Value
Number of patients	**81**
Female	28 (35%)
Male	53 (65%)
Age (years)	64 ± 10
BMI	
Normal (BMI < 25)	41 (51%)
Overweight (25 < BMI < 30)	27 (33%)
Obese (BMI > 30)	13 (16%)
TNM (7th edition)	
T2/T3/T4	12/41/28
N0/N1/N2	35/28/18
M0/M1	62/19
Treatment	
No treatment	1 (1%)
Surgery alone	37 (46%)
Neoadjuvant treatment	35 (43%)
Radiation 2 Gy × 25 with concomitant chemotherapy	29 (82%)
Radiation 5 Gy × 5 with sequential chemotherapy	6 (17%)
Palliative chemotherapy	8 (10%)
Survival	
PFS events	38 (47%)
OS events	43 (53%)

Data are number of patients, with percentages in parantheses, or mean ± standard deviation.

BMI = body mass index, TNM = tumor node metastasis, PFS = progression-free survival, OS = overall survival.

### Magnetic resonance imaging

MRI was performed on a Philips Achieva 1.5 T system (Philips Healthcare, Best, The Netherlands). In addition to routine T2-weighted high-resolution fast spin echo (T2w) images, an extended echo-planar imaging based DW MRI sequence with seven b-values of b = 0, 25, 50, 100, 500, 1000 and 1300 s/mm^2^ and a dynamic multi-echo contrast MRI sequence with three echoes with echo times (TE) = 4.6, 13.9 and 23.2 ms was collected. The latter was acquired using a split dynamic acquisition^9^, using a bolus injection of 0.2 ml/kg body weight of Dotarem® (279.3 mg/ml, Guerbert Roissy, France) directly followed by a 20 ml saline solution. Further details regarding the image acquisitions are found in Supplementary Table S1 and in^11,17^. To reduce bowel movement, glucagon (1 mg/ml, 1 ml intramuscularly) and Buscopan® (10 mg/ml, 1 ml intravenously) were administered before scanning. The Buscopan® injection was repeated before the acquisition of the dynamic images.

### Image pre-processing

While the T2w images were z-score normalized and used directly as input to the radiomics analysis, voxel-wise quantitative parameter maps were calculated from the DW and multi-echo MR images. For the DW images, the apparent diffusion coefficient (ADC) was calculated using the standard mono-exponential method^21^. From the dynamic multi-echo contrast MRI sequence, both T1w DCE- and T2*w DSC curves were extracted, which were further processed to parameter maps. Details regarding this have been published previously^10^. In brief, the DCE curves were fitted to an extended Tofts model^22^ for estimation of the volume transfer constant (K^trans^), the rate constant (k_ep_), the plasma volume (v_p_) and the extravascular extracellular volume (v_e_). Based on previous studies^9,10^, the K^trans^ parameter has shown the highest prognostic potential and was used as input to the radiomics analysis. For the DSC analysis, the T2*w signal was used in a model-free approach^23^ for estimation of the perfusion parameter BF and area under the curve (AUC). The BF is calculated by deconvolution of the contrast curve with the arterial input function. The AUC is a model free description of the contrast enhancement curve. The analysis of dynamic multi-echo data was done in NordicICE, version 4.0 (NordicNeuroLab, Bergen, Norway) and analysis of DW data was done in Python v3.7. The T2w images and the ADC, K^trans^, BF and AUC maps were used as input to the radiomics analysis.

### Tumor volume definition

We have previously developed methods for semi-automatic and automatic tumor volume definition^17,18^. This allowed us to explore whether the method of tumor volume definition affect radiomics feature extraction and their correlation to clinical outcome in this analysis. Tumor volumes defined by three different methods were used; (1) manually by two radiologists (Manual-A and Manual-B) with 14 and 7 years of experience with abdominal MRI delineating the tumor volume on the T2w images with DW images available as guidance where the median interobserver per patient Sørensen Dice similarity coefficient (DSC_P_) is 0.87 (interquartile range (IQR): 0.07) as described in^18^; (2) semi-automatically based on classical machine learning using voxel-wise classification via adaptive boosting (ADA) combined with morphological postprocessing^17^. This model was trained on raw image data from the T2w, DW, and dynamic multi-echo MRI of the same cohort and reported a DSC_P_ of 0.72 (IQR: 0.16)^17^. And (3), volumes which were automatically segmented using a DL 2D U-Net trained on the T2w and b500 DW images, giving a DSC_P_ of 0.77 (IQR: 0.14), as described in^18^.

### Feature extraction

Radiomic features were extracted using Pyradiomics v2.2^24^ in Python v3.7, see Supplementary material Appendix A1 for the configuration file. Features were extracted for the T2w images and the four parameter maps (ADC, K^trans^, BF and AUC). The extraction was repeated with all four tumor volume definitions (Manual-A, Manual-B, Automatic, Semi-Automatic). The tumor masks were originally defined on the T2w image grid, and rigid image registration was used to transfer the masks to the corresponding parameter map coordinate system.

For each combination of image and volume definition, 94 features were extracted. These features can be classified into six feature families; first order statistics (FO, n = 19), gray level cooccurrence matrix (GLCM, n = 24), gray level run length matrix (GLRLM, n = 16), gray level size zone matrix (GLSZM, n = 16), gray level dependence matrix (GLDM, n = 14) and neighboring gray tone difference matrix (NGTDM, n = 5). Detailed extraction configurations can be found in the Supplementary Appendix  A1, and the exact definitions of the individual features can be found in the Pyradiomics documentation. For the feature extraction, the bin width parameter (binWidth) was adjusted for each image type to split the intensity range of the corresponding image into approximately 100 bins.

### Assessment of feature stability

To assess the stability of each radiomics feature from the different image inputs under the varying tumor volume definitions, the interclass correlation coefficient (ICC) was calculated. The two-way random effects, absolute agreement, single rater ICC(2,1) was used, following the definition in^25^:

$$ICC\left(\mathrm{2,1}\right)= \frac{{MS}_{R}- {MS}_{E}}{{MS}_{R}+\left(k-1\right){MS}_{E}+ \frac{k}{n}({MS}_{C}- {MS}_{E})}$$

where rater here stands for the tumor volume definition, ${MS}_{R}$ is mean square for rows (patient samples), ${MS}_{E}$ is mean square for error (average extent to which the rater’s scores equal), ${MS}_{C}$ is mean square for columns (raters), k is number of raters and n the number of subjects.

Following the same guidelines, the reliability of the radiomics features was classified as poor (ICC < 0.5), moderate (0.5 <  = ICC < 0.75), good (0.75 <  = ICC < 0.9) or excellent (0.9 <  = ICC). In the subsequent analysis identifying radiomics features with prognostic potential, only features with excellent reliability were included, derived from the manual tumor contour of the most experienced radiologist (Manual A). The calculation of ICC(2,1) was performed with pingouin v0.4^26^ in Python v3.7.

### Statistical analysis

Three models (clinical, radiomics and combined clinical and radiomics) were made to identify whether clinical variables and/or the stable radiomics features could predict the patients’ outcome. Univariable and multivariable Cox regression were performed to find significant predictors. Considering the small number of events, a maximum number of two candidate predictors were used to avoid overfitting. The predictors with the highest hazard ratio (HR) in univariable analysis and no significant correlation (using the Spearman correlation test) were chosen to build the radiomic model. Selected predictors in the clinical and radiomic models were used to build the combined model. Internal validation was performed on 1000 bootstrap resamples to estimate the optimism-corrected area under the receiver operating characteristic curve (AUC). The Youden index was used to determine the threshold for calculating the sensitivity, specificity, positive predictive value (PPV), negative predictive value (NPV) and accuracy. The Youden index is defined as sensitivity (%) + specificity (%) − 100. Sensitivity (%) is defined as $\frac{true\,positives (TP)}{TP +false\, negatives (FN)}$ and specificity (%) as $\frac{true \,negatives (TN)}{TN +false \,positives (FP)}$, whereas PPV is defined as $\frac{TP}{TP+FP}$, and NPV as $\frac{TN}{TN+FN}$.

Calibration, which presents the agreement between the actual outcomes and predicted probabilities, was evaluated using the scatter plot where the x = y line indicates perfect calibration. Decision curve analysis (DCA) was performed to visualize the net benefit of models considering “treat none” and “treat all” as the benchmarking strategies. The net benefit was calculated as a function of relative harms related to the false predictions for each threshold of predicted probability. A nomogram presenting a sample patient was developed to improve the interpretability and reusability of the prediction models. Variables were compared by the Mann–Whitney U test, Chi-square test or Fisher’s exact test. Differences in survival were assessed by the log-rank test and visualized with Kaplan–Meier plots based on separating patients in two groups over/under the optimal cutoff of the variable presented. All tests were two-sided. A *p*-value < 0.05 was considered statistically significant. Analyses were performed in R v.4.1.2 (R Foundation for Statistical Computing) or in Python v3.7.

## Results

### Feature extraction and stability analysis

**Figure 3:**
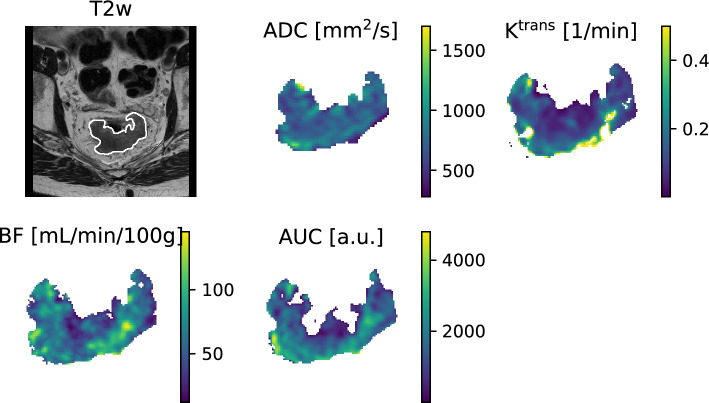
Illustration of images used as input to the radiomics analysis. The T2-weighted (T2w) magnetic resonance (MR) image is shown together with the tumor contour made by the more experienced radiologist. In addition, the quantitative parameter maps for the apparent diffusion coefficient (ADC), plasma transfer constant (K^trans^), blood flow (BF) and area under the curve (AUC) derived from the dynamic susceptibility contrast (DSC) MR images are shown.

Figure 3 shows a T2w image and the ADC, K^trans^, BF and AUC maps for one patient, as an example of the input to the radiomics analysis. From each of the five input images, 94 different radiomics features were extracted.

**Figure 4:**
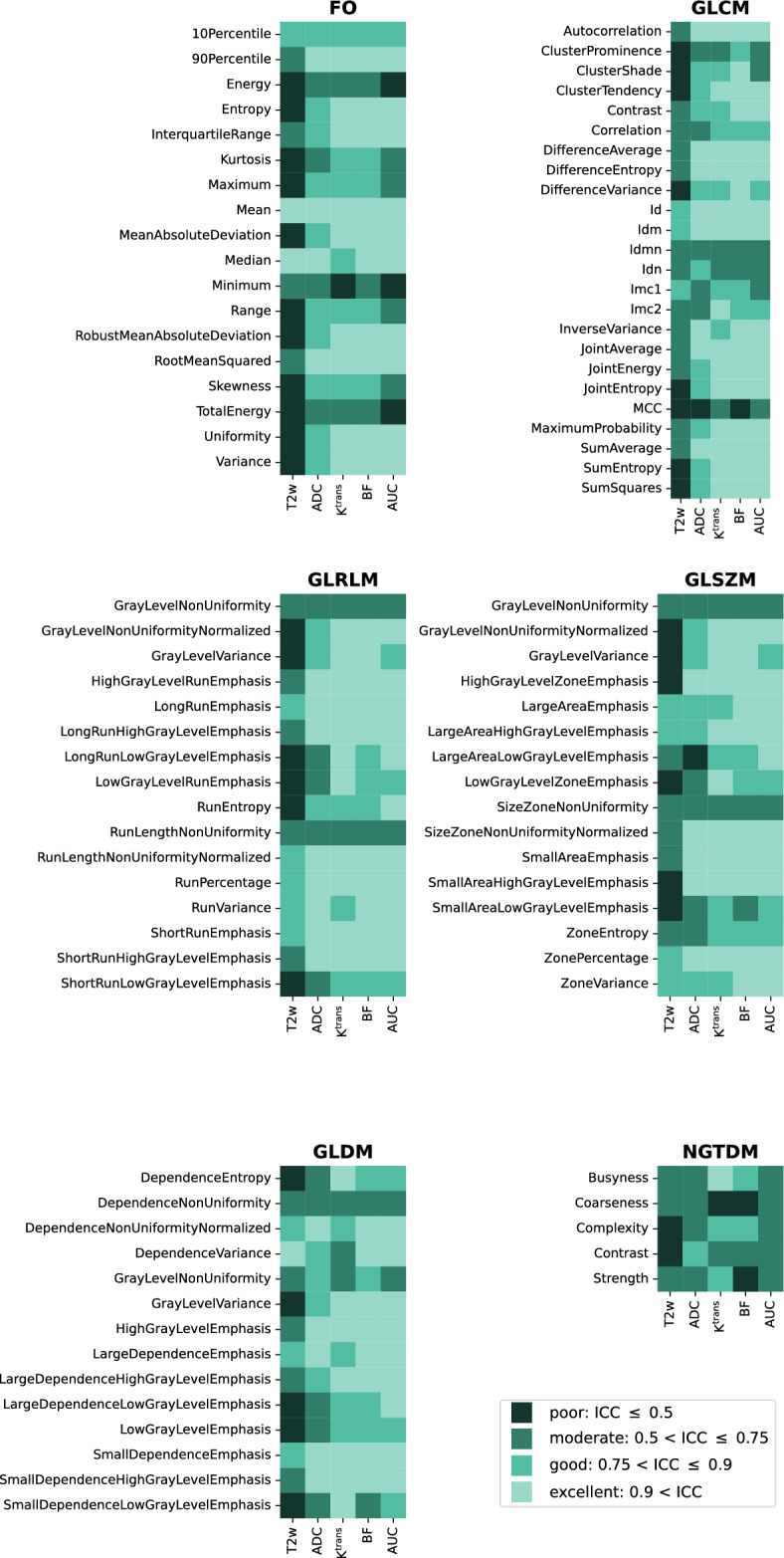
Result of the stability assessment of the radiomics feature values under contour variation within the six feature families for the T2-weighted (T2w) magnetic resonance image (MRI) and the 4 quantitative MRI maps, based on the interclass correlation coefficient (ICC), ICC(2,1) value. FO: first order, GLCM: gray level cooccurrence matrix, GLRLM: gray level run length matrix, GLSZM: gray level size zone matrix, GLDM: gray level dependence matrix, NGTDM: neighboring gray tone difference matrix, ADC: apparent diffusion coefficient, K^trans^: plasma transfer constant, BF: blood flow, AUC: area under the curve.

Figure 4 shows a list of all these features separated into the six different feature classes. The color maps show the distribution of the mean ICCs over all patients, which were calculated based on the four different volumes as a means to identify the stability of the features when the volume is varying. Overall, the features from the T2w images had a low ICC. Oppositely, the features from the qMRI parameters, in particular K^trans^ and BF, showed high ICC. The Supplementary Figure S2 shows a more detailed overview of the ICC per feature and image type.

**Figure 5:**
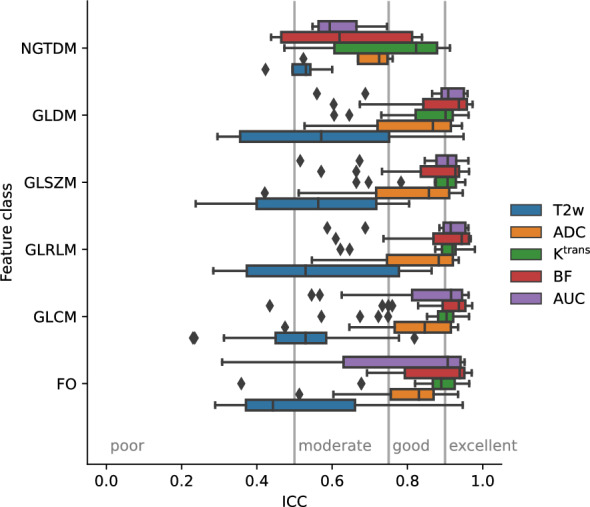
Overview of the interclass correlation coefficient (ICC), ICC(2,1) values, as measurement of feature stability under contour variation for the different feature classes and the different images and parameter maps used as input. FO: first order, GLCM: gray level cooccurrence matrix, GLRLM: gray level run length matrix, GLSZM: gray level size zone matrix, GLDM: gray level dependence matrix, NGTDM: neighboring gray tone difference matrix, ADC: apparent diffusion coefficient, K^trans^: plasma transfer constant, BF: blood flow, AUC: area under the curve.

In Fig. 5, the ICC is summarized per feature class and image type, confirming the low ICC with high variation in features from T2w images and high ICC with low variation in features from K^trans^ and BF. Five of the six feature classes presented very similar trends, where the performance of the last class (NGTDM) was inferior to the others. In order to select stable and robust features for the outcome analysis, we set the cutoff at 90% ICC, i.e., excellent reproducibility.

### Outcome analysis

Based on the clinical parameters (Table 1) and the identified stable radiomics features with ICC > 90%, outcome prediction models consisting of either clinical or radiomics features or a combination, were developed for both PFS and OS. The clinical parameters age, sex, body mass index (BMI), T stage and N stage were candidate parameters to the clinical prognostic model (Table 2A and B). The two parameters that were statistically most different between the groups were selected. In univariable analysis, sex and T stage were found to have significant associations with PFS, and BMI and T stage showed significant associations with OS.

**Table 2:** Descriptive statistics for (A) progression/no progression and (B) survival/no survival.

(A)			
Characteristic	No progression (n = 40)^1^	Progression (n = 22)^1^	*p*-value^2^
Age (years)			0.70
< 66	20 (50%)	10 (45%)	
≥ 66	20 (50%)	12 (55%)	
Sex			0.07
Female	14 (35%)	3 (14%)	
Male	26 (65%)	19 (86%)	
Body mass index			0.7
Normal	20 (50%)	9 (41%)	
Overweight	14 (35%)	8 (36%)	
Obese	6 (15%)	5 (23%)	
T stage			0.019
T2	7 (18%)	4 (18%)	
T3	26 (65%)	7 (32%)	
T4	7 (18%)	11 (50%)	
N stage			0.7
N0	22 (55%)	10 (45%)	
N1	12 (30%)	9 (41%)	
N2	6 (15%)	3 (14%)	
Time to progression (months)	69 (59, 84)	18 (10, 29)	< 0.001

(B)			
Characteristic	Survivor (n = 47)^1^	Non-survivor (n = 15)^1^	*p*-value^2^
Age (year)			0.5
< 66	24 (51%)	6 (40%)	
≥ 66	23 (49%)	9 (60%)	
Sex			0.5
Female	14 (30%)	3 (20%)	
Male	33 (70%)	12 (80%)	
Body mass index			0.2
Normal	24 (51%)	5 (33%)	
Overweight	17 (36%)	5 (33%)	
Obese	6 (13%)	5 (33%)	
T stage			0.025
T2	8 (17%)	3 (20%)	
T3	29 (62%)	4 (27%)	
T4	10 (21%)	8 (53%)	
N stage			> 0.9
N0	25 (53%)	7 (47%)	
N1	15 (32%)	6 (40%)	
N2	7 (15%)	2 (13%)	
Time to overall survival (months)	69 (59, 84)	29 (16, 42)	< 0.001

^1^ Values are presented as n (%) or median (IQR).

^2^ Tested by Mann–Whitney U test, Chi-squared test, or Fisher’s exact test.

Abbreviations: BMI: body mass index; IQR: interquartile range.

Table 3A and B shows the results from the clinical model, the radiomics model, and the combined model using the best clinical and radiomics parameters as input. For the analysis against PFS (Table 3A), sex was most important for the clinical model, whereas for the radiomics model the best feature was a feature based on the K^trans^ parameter, the FO mean absolute. In the combined model, the feature based on K^trans^ remained as the most significant variable, with an HR of 1.63 (95% CI = 1.05–2.52, *p* = 0.03). For the analysis against OS (Table 3B), high BMI was important for the clinical model, whereas for the radiomics model the best feature was a feature based on the BF parameter, the BF GLCM id parameter. In the combined model, the feature based on BF remained as the most significant variable, with an HR of 2.16 (95% CI = 1.18–3.95,* p* = 0.013).

**Table 3:** Clinical, radiomic and combined models for prediction of (A) progression-free survival (PFS) and (B) overall survival (OS).

(A)			
	HR	95% confidence interval	*p*-value
Clinical model			
Male sex (reference = female)	3.27	0.96–11.14	0.06
T stage (reference = T2)			
T3	0.52	0.15–1.78	0.3
T4	2.27	0.72–7.21	0.2
Radiomic model			
K^trans^, FO, Mean Absolute	1.87	1.18–2.97	0.008
ADC, GLCM, Joint Average	1.79	1.11–2.9	0.02
Combined model			
Male sex (reference = female)	2.41	0.71–8.25	0.2
K^trans^, FO, Mean Absolute	1.63	1.05–2.52	0.03

(B)			
	HR	95% confidence interval	*p*-value
Clinical model			
Body mass index (reference = normal)			
Overweight	2	0.53–7.47	0.3
Obese	3.15	0.90–11.02	0.07
T stage (reference = T2)			
T3	0.38	0.09–1.73	0.2
T4	1.8	0.43–7.47	0.4
Radiomic model			
BF, GLCM, ID	2.07	1.16–3.69	0.013
K^trans^, FO, Mean Absolute	1.92	1.15–3.21	0.013
Combined model			
Body mass index (reference = normal)			
Overweight	1.45	0.41–5.06	0.6
Obese	3.57	0.99–12.86	0.05
BF, GLCM, ID	2.16	1.18–3.95	0.013

PFS: progression free survival; OS: overall survival; HR: hazard ratio; K^trans^: volume transfer constant; FO: first order; ADC: apparent diffusion constant; GLCM: gray level co-occurrence matrix; BF: blood flow; ID: inverse difference.

**Figure 6:**
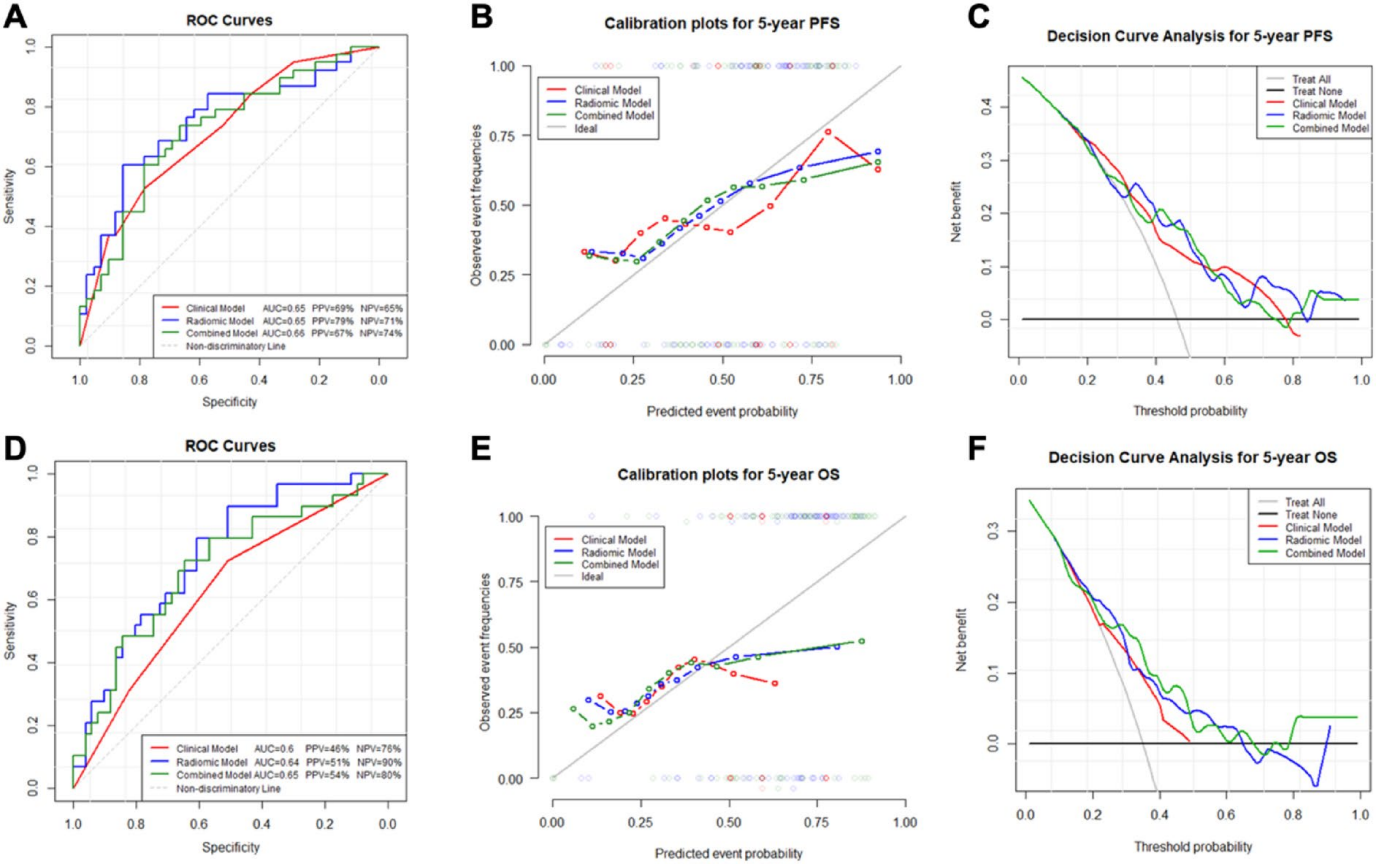
Receiver operative curve (ROC) analysis for progression-free survival (PFS) and overall survival (OS) (**A** and **D**), calibration plots for PFS and OS (**B** and **E**), and decision curve analysis (DCA) (**C** and **F**) for PFS and OS for the clinical model alone (red), radiomics model alone (blue) and the combined clinical and radiomics model (green).

Figure 6 shows the ROC analysis, calibration and DCA for PFS and OS for the three models. The models perform relatively similar, although for PFS the radiomics model had the highest AUC of 68.0% (standard deviation (SD): 19%), with a PPV of 51% and a NPV of 95%, and for OS the combined model had the highest AUC of 69.4% (SD: 22%), with a PPV of 46% and NPV of 92%. In the calibration plots, it can be seen that for the PFS the clinical model underestimates the actual risk, whereas for the OS all three models performs quite similar, although the clinical model underestimates the risk for high event probabilities. In the DCA for PFS, it can be seen that the clinical model provides a more accurate prediction compared to the radiomics model for middle-risk patients (threshold probability 0.3–0.6). However, for high-risk patients (threshold probability 0.7–0.9), only the radiomics model provided predictions that were acceptable, since that was the only model showing net benefits above both benchmarking lines. The DCA of the models for OS showed similar results. However, for the high-risk patients the combined model with both radiomics and clinical input were better than the radiomics only model. Nomograms of the combined model for predicting the 5-year PFS and OS are shown in Supplementary Figure S3.

**Figure 7:**
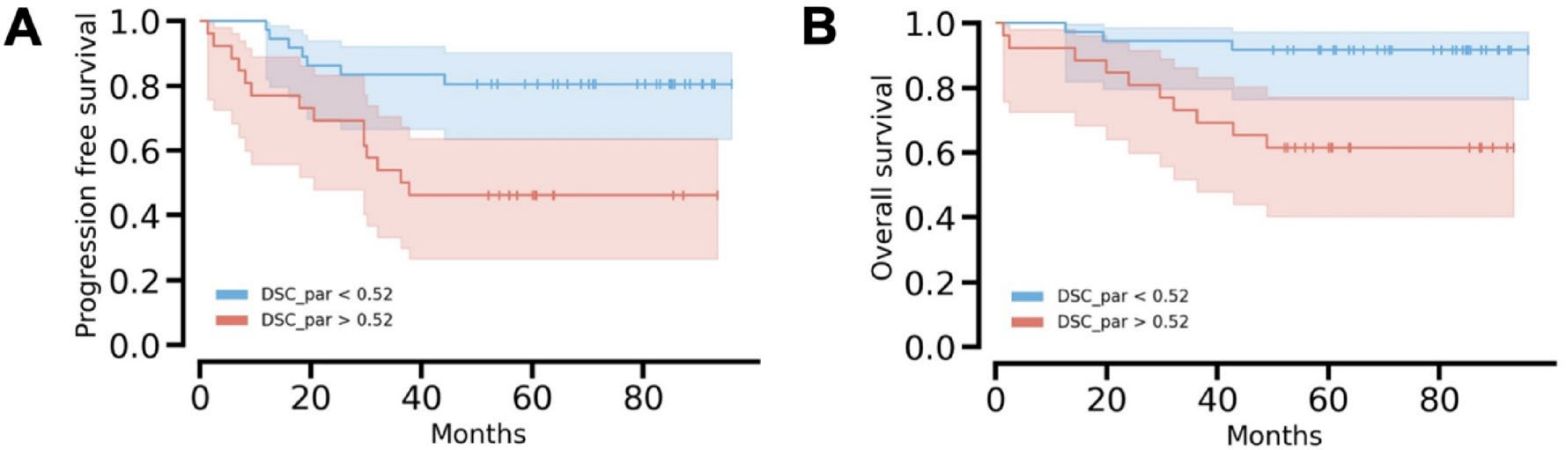
Kaplan–Meier plot for the blood flow (BF) gray level size zone matrix (GLSZM) id feature for progression-free survival (PFS) and overall survival (OS), when separating patients above and below the optimal cutoff. For PFS, the difference in progression at 60 months was 54% for the group of patients above the cutoff, and 19% for the group of patients below the cutoff. For OS, the survival difference at 60 months was 38% for the group of patients above the cutoff and 8% for patients below the cutoff. The log-rank tests for BF GLSZM id against resulted in *p* = 0.004 for PFS and *p* = 0.004 for OS.

A log-rank test with associated Kaplan–Meier plots were performed for the three radiomics features that were identified in Table 3 (K^trans^ FO mean absolute, ADC GLCM joint average, BF GLSZM id). When evaluated against both PFS and OS, the log-rank tests revealed that only the BF GLSZM id feature was significant (*p* = 0.004 for PFS and *p* = 0.004 for OS). Figure 7 shows the Kaplan–Meier plot for the BF GLSZM id feature for PFS and OS, when separating patients above and below the optimal cutoff. For PFS, the difference in progression at 60 months was 54% for the group of patients above the cutoff, and 19% for the group of patients below the cutoff. For OS, the survival difference at 60 months was 38% for the group of patients above the cutoff and 8% for patients below the cutoff.

## Discussion

In this study we aimed to identify radiomics features with prognostic potential from MR images of rectal cancer, which were stable against interobserver variability in tumor contouring. From the stable radiomics features, our main finding was that features based on qMRI contained higher prognostic potential than features based on high-resolution T2-weighted MRI sequences. Also, when the radiomics features were compared to clinical parameters, the qMRI radiomics features demonstrated the best prognostic potential, in particular the radiomics feature based on the DSC MRI parameter BF, BF GLSZM id, which was significantly associated with both PFS and OS.

Given that we had several input volumes available (two sets of manual contours, semi-automatic machine learning contours and deep-learning contours) we had the opportunity to identify which radiomics features were stable and robust across the different input volumes. By doing such a stability analysis, one may reduce the noise and probability of random findings in the feature extraction process. Previously, few studies have investigated stability of qMRI-based radiomics, however, two studies using the ADC from DW MRI exist. One study assessed the stability of ADC-based radiomics features in rectal cancer^27^, finding that shape features were strongly affected by delineation quality whereas reproducibility of textural features was poor when the image pre-processing was varied. In contrast, features from intensity distributions were less sensitive to variation in both pre-processing and delineations. A study by Peerling et al. showed low test–retest stability of ADC-based radiomics features in a multicenter study in lung cancer, ovarian cancer and liver metastases of colorectal cancer^28^. In addition, they showed that the feature stability was affected by the type of MRI scanner and the field strength of the scanner. In the stability analysis in our study, we show that relevant and stable radiomics features can be extracted from volumes defined by automatic deep learning algorithms. This represents an important step towards an automated workflow, which is essential if radiomics analysis is to be integrated as a clinical tool.

The stability analysis was conducted using the measure recommended by^25^, the two-way random effects, absolute agreement, single rater ICC(2,1) measure. We discovered that many T2w features were removed because their ICC was low, whereas the ICC results were overall higher and more consistent when qMRI was used as input, especially those based on the K^trans^ and BF parameters (Figs. 4 and 5). In rectal cancer, we have not found other studies using K^trans^ or BF parameter maps as input to radiomics analysis, but several papers used ADC as input. In a recent study with 898 patients with rectal cancer treated with CRT, T2w images and ADC were used as input to radiomics analysis for prediction of pathologic complete response^29^. The study showed that a model with both T2w images and ADC was inferior to only using T2w images alone. However, in a different study^30^ a pre-treatment qMRI radiomics model including features from ADC, T2w and DCE MRI was significantly associated with pathologic complete response. The authors recently published that the same model also was related to disease free survival^31^, and if clinical risk factors in terms of pathologic N-stage and tumor differentiation were included the model improved even further. In other cancer types, there are examples of other MRI information used as input to radiomics. In a study in breast cancer, a radiomics model based on features from T2w, diffusion kurtosis imaging and DCE MRI parameter maps showed high ability to discriminate benign and malignant breast lesions^32^. In addition to our stability analysis, we also believe that using a standardized radiomics pipeline (Pyradiomics) adds robustness to our results. Given the few studies that have addressed stability of qMRI radiomics features, we believe our approach may provide an example on how this can be conducted.

After we identified the robust radiomics features, their prognostic potential against 5-year PFS and OS was compared with the prognostic potential of common clinical parameters, as well as with a combined model based on both radiomics and clinical parameters. The models successfully predicted both PFS and OS. Interestingly, in the combined models, the radiomics features based on qMRI remained as the most significant (K^trans^ FO mean absolute for PFS and BF GLSZM id for OS) (Table 3A and B). Furthermore, the DCA analysis revealed that the radiomics features were important in predicting both PFS and OS for high-risk patients (Fig. 6). For PFS, the radiomics model alone was most valuable, whereas for OS the combined radiomics and clinical model was the most optimal. The result that the clinical model alone provides inferior predictions for high-risk patients, provides support for including radiomics features into clinical decision support. In line with this finding, it was previously found in the same patient cohort showed that the K^trans^ and BF parameters are important. In one study we found that K^trans^ was associated with higher probability of lymph node metastasis^9^, whereas the BF parameter has been associated with both CRT response and OS^11^. The finding that these parameters remain significant also in a radiomics study, supports that these parameters are stable and important markers to assess and predict the development of rectal cancer progression.

Ideally, we should have validated our results in independent cohorts. However, since our study-specific DCE MRI and DSC MRI sequences are not commonly acquired in rectal cancer, we were unable to find suitable validation cohorts. This highlights the need for standardization in both image acquisition and post-processing of qMRI^13^ in order to achieve validated models that can qualify for clinical integration^33^. Furthermore, MRI sequences not requiring intravenous contrast agents are preferable due to storage of gadolinium in the brain. However, these patients have a life-threatening disease and the addition of a gadolinium-based contrast agent is justified.

## Conclusion

We provide an approach to identify stable qMRI-based radiomics features with prognostic value. In particular, a radiomics feature based on BF from DSC MRI was stable and associated with both PFS and OS. qMRI as input to radiomics for robust outcome analysis is novel, but further studies are needed to fully identify the potential benefit of qMRI for radiomics.

### Supplementary Information

Supplementary Information.

## Data Availability

The datasets generated and analyzed during the current study are available from the corresponding author upon reasonable request.
