# Supplementary material for: Quantitative MRI-based radiomics analysis identifies blood flow feature associated to overall survival for rectal cancer patients
Source: Sci Rep. 2024 Jan 2;14:258. doi: 10.1038/s41598-023-50966-9 (PMC10762039; doi:10.1038/s41598-023-50966-9)
Supplement: Supplementary file 1 — Supplementary Information. [file 41598_2023_50966_MOESM1_ESM.pdf]

Supplementary information

Supplementary Table S1. Overview of acquisition parameters used in the different MRI sequences.

**Image sequence**	**T2w**	**T2*w**	**DW**	**DME**
Sequence	FSE	FFE	2D EPI	3D EPI
Repetition time / s	2.82–3.04	9.49	3	0.38
Echo time / ms	80	4.6, 13.8, 23.0, 32.2, 41.4	75	4.6, 13.9, 23.2
Averages	6	3	6	1
Acquisition matrix	256/254	180/120	80/60	92/90
In plane resolution / mm	0.35	0.70	1.25	0.70
Slice thickness / mm	2.50	3.00	4.00	10
Slice separation / mm	2.75	4.00	4.30	5
Scan time† / min	7	6	8	7

T2w: T2-weighted; T2*w: T2*-weighted; DW: diffusion weighted; DME: dynamic multi echo; FOV: field of view; FSE: fast spin echo; EPI: echo planar imaging; FFE: steady state gradient echo; †: median values, dependent on number of imaged slices.

Supplementary Figure S2. The figure shows the interclass correlation coefficient (ICC), ICC(2,1) value, and the corresponding confidence interval, for the features based on the different images. The dashed lines indicate the limits used to bin the ICC values into categories (poor, moderate, good, excellent). We refer to the Pyradiomics documentation for details regarding the definition of the individual features.

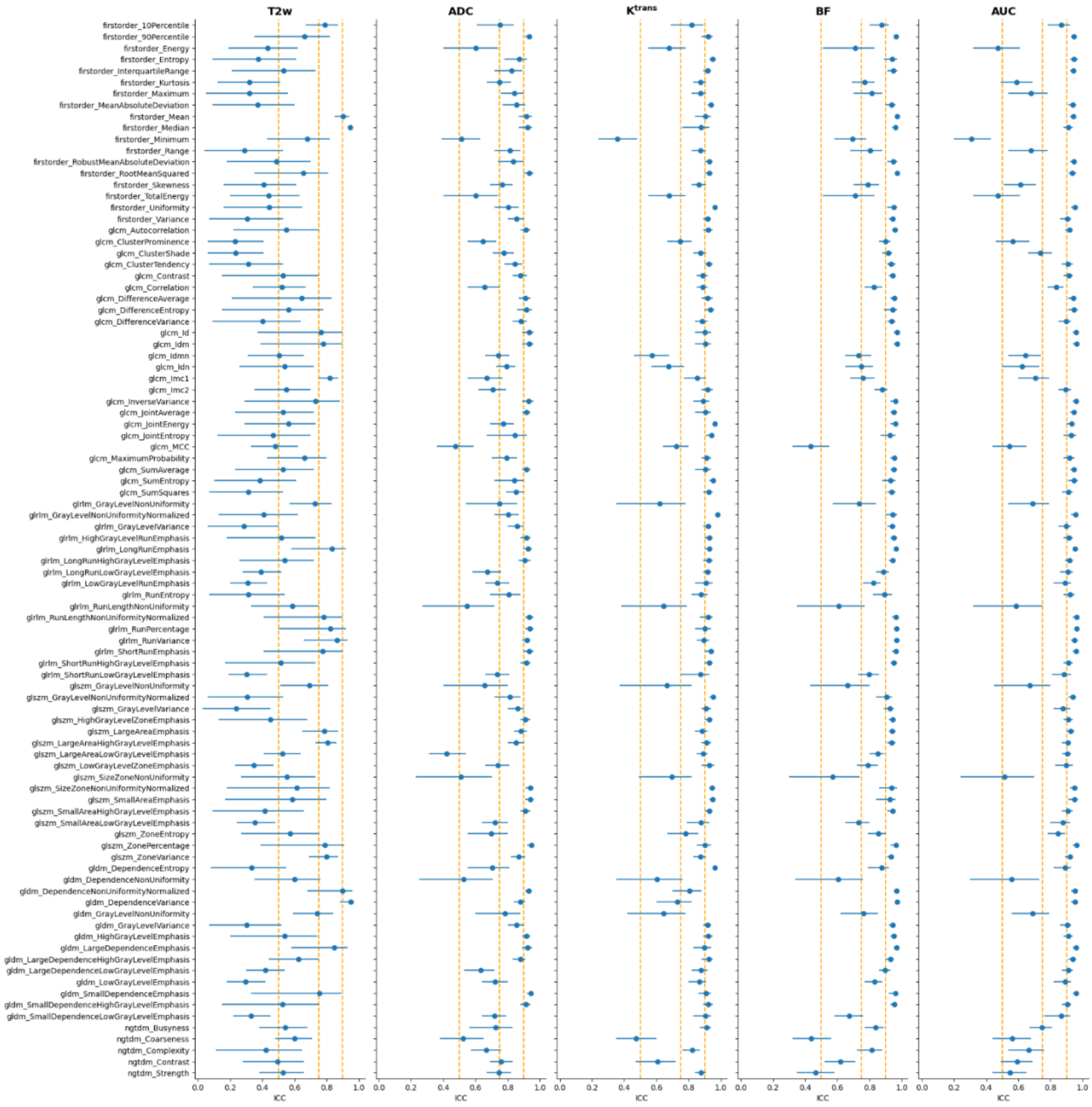

Supplementary Figure S3. A nomogram of the combined model for predicting the 5-year progression free survival (PFS) for a sample patient, using the IDCEKtrans original first order mean absolute feature and sex as input (upper figure). A nomogram of the combined model for predicting the 5-year overall survival (OS) for a sample patient, using the IDSCBF original glcm inverse different feature and body mass index as input.

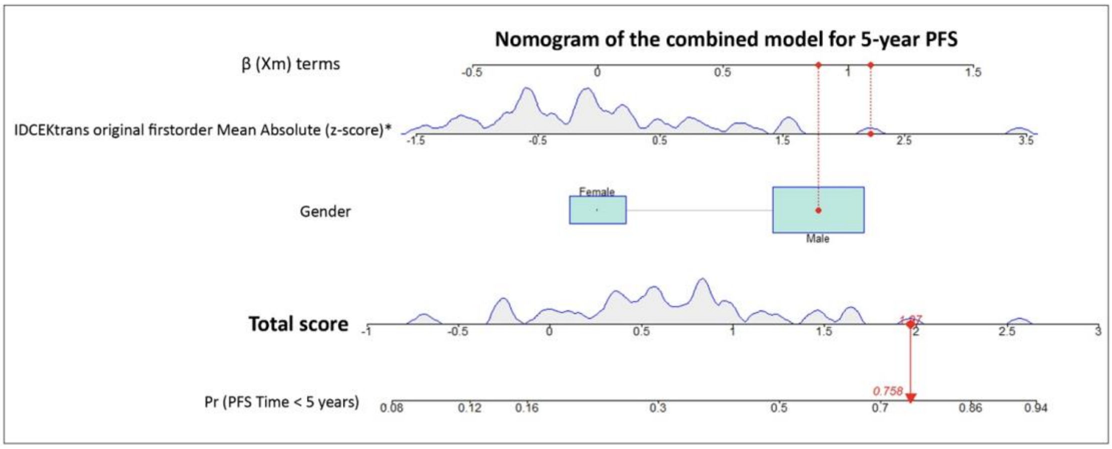

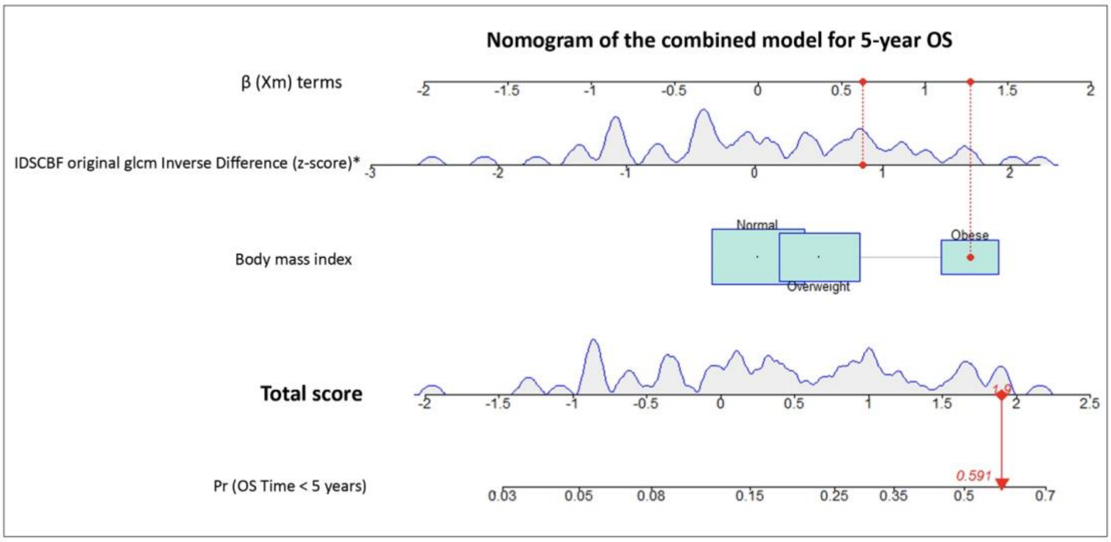

Supplementary Appendix A1: Pyradiomics feature extraction configuration

Feature extraction was performed using Python v.37 and the pyradiomics v.2.2 package.
If not otherwise specified, pyradiomic default values were used. The different yaml files for the feature extraction from the different image and parameter maps were combined into one for readability.

```
imageType:
        Original: {} # unfiltered image

featureClass:
          # calculate all features (excluding redundant/deprecated features).
          firstorder: []
          glcm: []
          glrlm: []
          glszm: []
          gldm: []
          ngtdm: []

setting:
          geometryTolerance: 0.001
          force2D: True

          #T2w specific settings:
          binWidth: 0.05
          normalize: True

          # ADC specific settings
          binWidth: 30
          normalize: False

          # K_trans specific settings
          binWidth: 0.025
          normalize: False

          # BF specific settings
          binWidth: 7
          normalize: False

          # AUC specific settings
          binWidth: 150
          normalize: False
```

T2w: T2-weighted; ADC: Apparent diffusion coefficient; K_trans: Plasma transfer constant; BF: Blood flow; AUC: Area under the curve.
